# Reducing the Burden of Mortality in Older People With Diabetes: A Review of Current Research

**DOI:** 10.3389/fendo.2020.00133

**Published:** 2020-03-17

**Authors:** Angus Forbes

**Affiliations:** Clinical Diabetes Research, King's College London, London, United Kingdom

**Keywords:** diabetes, older people, mortality, risk factors, glycemic control

## Abstract

This review considers the burden of mortality observed in the older population of people with diabetes and identifies the risk factors associated with mortality hazard in this population. The mortality gap between older people with and without diabetes is enduring, with excess mortality being 10% greater than in the general population. While early mortality in men with diabetes is significantly greater than females with diabetes, the relative mortality risk in females is much higher compared to women without diabetes. Older people who have developed diabetes in middle age have significantly higher mortality hazard compared to those who develop it in old age, emphasizing the continued importance of optimizing diabetes care in all ages. To minimize mortality hazard in older age it is important to address some of the factors that convey risk, these include: comorbidity; polypharmacy; physical and mental frailty; safe glycemic targets for older people; hypoglycemia; glycemic targets; and the hypoglycemic agents. While the data to determine optimal management approaches are limited, the overall need is for a more diligent approach in assessing the needs of older people with diabetes to inform individualized care strategies and therapy goals that minimize potential hazards.

## Introduction

Despite overall improvements in the life-expectancy for people living with diabetes in recent years, there is still a significant disparity in the mortality hazard for people with diabetes compared to those without (1–5). This disparity continues throughout the lifespan and while mortality hazards naturally increase with advancing years, the factors associated with this risk in older people with diabetes become more complex and include: diabetes related tissue damage and complications; polypharmacy; co-morbidity; and mental and physical frailty (6–11). In addition, there is a growing recognition that the traditional diabetes care model of intensifying hypoglycemic therapies to reduce glucose exposure may not always be appropriate in managing diabetes in older age. There has been some concern over: the use of some glucose lowering medications in the older population with diabetes, particularly sulfonylureas and insulin (12–15); and in setting appropriate levels for glycemic control (11, 16–20). In older age, it is equally important to consider quality of life and the need to promote physical, social, and psychological functioning. It also needs to be recognized that there is a high level of heterogeneity in the older population of people with diabetes and that chronological age is not a good discriminator in making care decisions. In addition, some older people particularly those who are frailer will have other care deficits that require the support of relatives or formal carers, including support with diabetes self-management and related areas, such as: nutrition; attending health care and screening visits; and skin care. In those with a high level of mental or physical impairment this may be in the context of a residential care or nursing home.

Hence, minimizing mortality hazard in older people with diabetes can be challenging and demands a very thoughtful and individualized approach when considering care objectives. Given that older people constitute a large and growing segment of the diabetes population, finding strategies to minimize hazards and enhance quality of life for old people is an important priority. A significant hindrance to this endeavor, is the lack of clinical studies involving older people. This limits the evidence available to clinicians in making informed decisions in respect of diabetes care in older people. The paper seeks to address this gap, by presenting a review of some of the factors associated with mortality in older people, to inform potential strategies that may ameliorate this risk. The review addresses the following areas:

- Mortality risk in older people with diabetes.
- Risk factors for excess mortality.
- Glycemic control, diabetes therapies and mortality.

### Mortality Risk

Studies comparing people with and without diabetes show that diabetes is associated with excess mortality (21, 22). In a recent UK retrospective cohort study, with 10 years follow-up of people aged ≥ 70 years with diabetes (*n* = 35,717) and without diabetes (*n* = 307,918), survival at 5 and 10 years was 8 and 11% lower, respectively, with an overall mortality hazard of 1.29(95%CI = 1.26–1.31), with a non-diabetes reference population (11). This study also showed that the relative risk of mortality was greater in females (HR 1.36; 95%CI = 1.33–0.140), although the absolute risk for premature mortality was higher in males, compared to people without diabetes. This gender difference was also reported in a study comparing mortality risk in older people with (*n* = 3,914) and without diabetes (*n* = 7,188) which found the relative mortality risks in males and females were elevated by 9 and 25%, respectively compared to the control population (23).

A further distinction in the mortality burden in older people with diabetes, can be made in respect of the duration of diabetes. The population can be broadly segmented into those who entre older age with diabetes having developed it in their middle years; and those who acquired diabetes in older age. Half of the older population of people with Type 2 diabetes develop diabetes after the age of 65 years (24). Type 2 diabetes developed in older age can often have different metabolic features compared to diabetes developed in the mid years and this population has a much shorter exposure to hyperglycemia (25). A systematic review and metanalysis of mortality considering diabetes duration, reported that the relative risk for mortality in men diagnosed between the ages of 60 and 70 was 38% greater than in those without diabetes, compared to 13% for those diagnosed aged 70 years or older (21). The review reposted a similar pattern for women, with relative risks 40 and 19% for the early and later diagnosed cohorts, respectively, compared to women without diabetes. A recent cohort study of people aged >70 years, reported showed that those who had diabetes for >10 years had a 37% higher risk of mortality compared to those with a duration of >3 years (11).

Therefore, we can see that despite longevity in both the diabetes and non-diabetes populations increasing, risk of mortality remains elevated in the diabetes population. Hence, it is important that we try to identify the factors that may contribute to this risk, so we can develop care approaches that will extend both the quantity and quality of life in older people with diabetes. The mortality data also highlight the inherent heterogeneity in the older diabetes population, with these variations indicating that there are different types of risks within the population. This would suggest the need for more sensitive care models that can help clinicians identify and respond more appropriately to the needs of the older person.

### Risk Factors for Excess Mortality

There are multiple potential risk factors that explain the excess mortality observed in older people with diabetes. Clearly the pathophysiological damage associated with diabetes resulting from the glucotoxic and lipotoxic environment that converge in diabetes, is the primary driver of mortality. As highlighted in the previous section, this is evident in the burden of mortality being associated with disease duration. This is also reflected in the causes of death in people with diabetes, with vascular disease, particularly cardiovascular disease (CVD) being the most common (26). All of which emphasizes the ongoing importance of achieving optimal metabolic control. However, in older people with diabetes there are potentially some additional risk factors that may be important, such as comorbidity, polypharmacy, and frailty.

While multi-morbidity is common in older age (27), some co-morbidities including dementia, depression and CVD are more prevalent in older people and bring with them enhanced mortality risk (7, 8, 10). As such these comorbidities are important in understanding mortality risk in this population, and that risk has been assessed in a number of studies. In a study of 750 older people with diabetes (mean age 69 ± 7). Laiteerapong et al. (27) clustered patients into three groups expressing comorbid load as: low (63%, *n* = 470), medium (29%, *n* = 215), or high (9%, *n* = 65). The relative mortality risks for these groups were 33, 17, and 9% higher the high, medium, and low groups, respectively. However, studies comparing the impact of comorbid load in people with and without diabetes have not indicated that the risk conferred by multiple comorbidities is any greater than for the general population (11). Hence, while multiple morbidities confer mortality risk to older people with diabetes, this may not be any more so than for older people without diabetes. Nevertheless, considering comorbidity in the management of diabetes in the older person remains important in terms of: minimizing any progression of the comorbidity; minimizing risks from multiple therapies; and considering the older persons preferences in respect of how these are managed to enhance functionality and quality-of-life.

While CVD is considered to be the greatest driver of mortality risk in people with diabetes (4, 10, 17), the risk conveyed by CVD may be more complex in the older person. Studies suggest that in the older population of people with diabetes, CVD is a more significant risk factor in the younger old (<75 years) (11, 28). Indeed, the fact that the lower effect of CVD on mortality in the older old, may be a survival phenomenon. Therefore, CVD prevention strategies should perhaps be a stronger consideration in the management of the younger old. Studies have also highlighted the importance of heart failure, which is more common in older people and has been associated with elevated mortality hazard compared to people without diabetes (11). Therefore, a higher vigilance of heart failure in older people with diabetes is required. In terms of treatment options, it may suggest that the SGLT2 group of drugs could be useful, as the trials have suggested reductions in heart failure and hospital admissions related to heart failure, although further studies to test these therapies in older populations are still needed (29, 30).

It is also known that some therapies, such as thiazolidinediones are associated with increased risk of heart failure (31) so should be avoided in older people at risk of or with heart failure. Diabetes complications also convey important risk *n* older people, particularly end-stage-renal failure and advanced foot complications. Hence, extra vigilance is required in respect of foot screening particularly as older people often present with more advanced tissue damage and in monitoring kidney function, noting changing trends in kidney performance rather than waiting to intervene when the disease is more advanced. Another important and overlooked comorbidity that has is common in older people with diabetes and has been associated with mortality is depression and assessing for and treating depression is another important consideration in this population (8).

Polypharmacy is a significant problem in older age and in diabetes in particular where people need to take multiple diabetes hypoglycemic therapies, antihypertensives, and lipid lowering therapies (32). Polypharmacy has been identified as conveying additional mortality hazard in older age (6, 33). In a large cohort study, Forbes et al. (11) they considered the mortality hazard in respective of older people taking 0–2, 3–4, 4–6, and >7 agents per day, and reported mortality risk elevations 7, 15, and 32% for the 3–4, 4–6, and >7 groups, respectively with 0–2 group as the reference. Although as was the case with comorbidities, the risk from polypharmacy in people with diabetes was lower than that observed in the non-diabetes population, this may be related to the nature of the therapies associated with diabetes, many of which aim to reduce complications, in contrast to other diseases, such as Parkinson's disease where therapies alleviate symptoms rather than regress the cause of the disease.

Frailty is being increasingly recognized as a significant risk moderator in older age. As a multifactorial condition associated with metabolic dysfunction, inflammatory processes and reduced physical capacity it clearly has a high reliance in the care of older people with diabetes. It has been estimated that frailty affects 30% of older people with diabetes (34). Studies have reported an increased mortality hazard in older people with diabetes and frailty (9, 35). A prospective cohort study of people aged ≥65 years with a 5 years follow-up of people with (*n* = 363) and without diabetes (*n* = 1,462) reported significantly higher mortality levels of frailty in those with diabetes, with frailty being the strongest predictor of mortality (19). Therefore, frailty and its associated deficits in metabolic function, nutritional intake and self-management capacity; is a consideration when making therapeutic choices for older people with diabetes (35). Hence, screening for frailty and possibly pre-frailty may help in risk management in this population. Consideration should also be given to nutrition in this group of patients, as frailty is also associated with a lower BMI which is an indicator of mortality risk (11, 36). Frailty, may also indicate that someone is approaching the end-of-life, emphasizing the need to promote quality of life and reduce treatment hazards as the focus of care provision (37, 38). Finally, strategies to prevent or delay frailly should be considered, there are some promising data indication that enhanced physical exercise regimens targeting those at risk and nutritional interventions may help reduce the impact of frailty (39).

### Glycemic Control and Hypoglycemic Therapies

It has long been established that hyperglycemia contributes significantly to mortality, and that more intensive control reduces mortality in diabetes (40) However, following the findings of recent trials of intensive glucose lowering, concerns have been raised in respect of the safety of the level of intensification and in the manner in which it is achieved (41–43). The ACCORD trial in particular was of concern, as it showed higher mortality in the intensified arm of their study, which involved a rapid (multiple therapies over 4 months) reduction in HbA1c to 6.4%. It has been suggested that the speed of intensification together with the levels of insulin and thiazolidinediones used compared to the control arm, may have heightened CVD risk following weight gain and excess hypoglycemia. The questions surrounding optimal levels of glycemia control in older people have been further complicated by a number of observational studies that have shown a J-shaped distribution of mortality hazard and glycemic control, with higher levels of mortality being observed in respect of both elevated and lower (HbA1c ≤ 7.0%) HbA1c values (16, 17). Collectively these trials and cohort studies have suggested that higher glycemic targets may be appropriate (44).

However, more recent population studies have suggested that assumptions in respect of glycemic control and mortality in older people may need to be reconsidered. A particular concern may be that over caution in respect of the lower levels of glycemia may underplay the importance of the higher levels. In a study of 1,279 adults with diabetes aged >65 years, Palta et al. (20), reported that all-cause mortality incrementally rose with increased HbA1c levels (with HbA1c <6.5% as a reference) by: 20% with HbA1c values from 6.5 to 7.9%; 60% with HbA1c 8–8.9%; and 80% in those with a HbA1c > 9%. Such data highlight the continued importance of reducing the glucotoxic burden to promote longevity in older people with diabetes. Hence, the overall picture on what might be an optimal target range for glycemic control in respect of mortality risk in older people, is somewhat unclear at present. Although the principle of setting individualized treatment parameters that reduce risks and promote quality of life is clearly the most appropriate approach for the older person.

Another recent cohort study of 54,803 people with diabetes aged >70 years, addressed both glycemic control and variability (11). In terms of glycemic control, while this study also observed a J-shape distribution for mortality risk and level of HbA1c, the threshold at which the hazard became significant at the lower leave of glycemic control was at a HbA1c of <6.0%. The study also considered variability (fluctuations) in glycemic control (based on changes of ≥0.5% over time) and found a linear relationship between the amount variability and mortality hazard. The hazard ratios in patients with the highest instability metric (*n* = 1,227) were 2.47 and 2.21 for females and males, respectively, with the lowest levels of variability group as the reference. The study reported that while in part this variability may be driven by diabetes therapies and perhaps over intensification; it also indicated that a falling HbA1c in the older population could be a marker for other physical changes, such as frailty (18), malnutrition (45), or hypoglycemia (20). Hence, the findings of this study suggest that variability in glycemic control and most notably a falling HbA1c not explained by a therapeutic intervention should be considered an important risk trigger a review of the older person's physical status and medications.

Finally, as previously indicated, a significant consideration in reducing the burden of mortality in older people with diabetes is the use of hypoglycemic therapies in diabetes. Hypoglycemia in both chronic and acute exposures, has been associated with excess mortality and in physical and cognitive degeneration (46, 47). In respect of diabetes therapies, insulin and sulphonylureas (SUs) have been identified as conveying mortality hazard in a number of observational studies (48). Although, in a more recent cohort study (previously mentioned) of people >70 years, insulin and SUs were only observed to contribute modestly to mortality risk (11). A Danish observational study comparing insulin and SU (*n* = 11,080) to insulin and metformin (*n* = 16,910) combinations reported an 80% higher mortality risk in the insulin + SU combination (49). While this study was not exclusive to older people it also made some useful distinctions between SU agents, showing that gliclazide was associated with a lower relative risk (RR) for mortality (RR = 1.5) compared to glibenclamide (RR = 2.0), glimepiride (RR = 1.79), glipizide (RR = 2.2), and tolbutamide (RR = 2.2). In combination with insulin gliclazide had a lower risk than metformin and insulin, although the confidence interval was quite broad (RR = 0.9, 95% CI: 0.6–1.5). These data reinforce the choice of shorter acting SUs in older people with diabetes. It is also important to emphasize that associations between insulin and SUs and mortality, is not necessarily cause and effect, rather it could be a reflection on the overall progression of the diabetes in individuals requiring these agents. Nevertheless, older people using insulin and SUs should be regularly reviewed, particularly in relation to hypoglycemia which may be under reported in older people as they can developed blunted awareness. Caution should also be executed in patients with a low HbA1c (<6.5%).

While the risks with insulin and SUs have been quite widely considered, less is known in respect of the mortality hazards of other agents in older people. Dipeptidyl peptidase-4 (DPP-4) inhibitors, offer a glucose lowering therapy with a reportedly low risk of hypoglycemia and they have been associated with less glycemic variability. However, they have also been associated with incident heart failure and may reduce appetite which may be inappropriate in some older people (50). A recent study considering incident hypoglycemia in older people (*n* = 5,130; mean age 73.8 ± 6.1) in relation to sitagliptin, reported no increase in hypoglycemia when sitagliptin was used as an adjunct to metformin but it did increase in association with SUs and insulin with respective odds ratios of 2.2 and 17.7 (51). GLP-1 receptor agonist have been studied in older people and show equivalent metabolic outcomes to younger people with diabetes (52), although there are no long-term mortality data for GLP-1s in older people and as with DDP-4 inhibitors they can reduce appetite and cause weight loss which would not be desirable in the older frail population. Meglitinides, which have a shorter duration of action compared to SUs might be an alternative, however, there are limited long-term data on diabetes complications and mortality on this group of drugs and a recent observational study of patients with advanced kidney disease showed increased risk of hypoglycemia (53). The newest agents to be added to the options for diabetes therapy are the sodium-glucose cotransporter 2 inhibitors (SGLT2-I), while the long-term mortality data for older people are limited they do show benefits for CVD outcomes including heart failure which may be important in treating older people (54, 55). However, they are also associated with genitourinary problems and could cause dehydration which may be hazardous in some older people. For the reasons previously outlined in respect of heart failure, thiazolidinediones are best avoided in older people, they also increase the risk of fractures which can be a very significant risk in older age.

Therefore, it is important to recognize that in older age reviewing therapy risks is of high importance. It may be that treatment regimens that were effective in someone's younger years are less appropriate and become more hazardous as they become older and their body changes. Hence, in addition to adding therapies to optimize diabetes control it becomes as important to consider deprescribing therapies, particularly where there is a risk of hypoglycemia. In-so-doing it is important to explain these reasons to the person and where appropriate their carers as this could potentially cause some anxiety. While evidence for optimal strategies to risk manage hypoglycemic therapies is limited, a Canadian group have produced a guideline based on a systematic literature review for deprescribing diabetes therapies in older age which provides a useful framework for risk minimization (56).

## Conclusion

Excess mortality in the older population of people with diabetes is an enduring concern. While this may be largely attributable to the pathophysiological damage associated with diabetes and its complications, this review has highlighted some potentially modifiable risk factors that could attenuate this hazard. Perhaps the most important consideration, is the need for clinicians to be more diligent in respect of assessing older people to individualizes care strategies and set therapy goals that minimize potential hazards. This will require new approaches to care that consider factors not generally considered in general diabetes management, these should include: assessments of physical and mental function; medicines review and risk assessment particularly in respect of hypoglycemia; nutritional status and BMI; and unexplained changes in glycemic control particularly a low HbA1c. Given the heterogeneity of the population, systems to stratify risk in older people may also be useful. An area not addressed in this review but which also needs to be considered in older age, is the interaction between the diabetes care provided and the carers and family members who support them. Finally, there is clearly a need for more research to identify optimal care strategies to reduce diabetes related risks in this population. While more trials are now including older people, there remains a significant deficit in the current evidence-base for this significant and important population.

## Author Contributions

The author confirms being the sole contributor of this work and has approved it for publication.

### Conflict of Interest

The author declares that the research was conducted in the absence of any commercial or financial relationships that could be construed as a potential conflict of interest.
